# Respiratory fit test panel representing population of Malaysia

**DOI:** 10.1186/s12890-024-02919-9

**Published:** 2024-03-07

**Authors:** Yin Cheng Lim, Shahrul Aiman Soelar, Ameerah Su’ad Abdul Shakor¹, Nadia Mohamad, Muhammad Alfatih Pahrol, Rohaida Ismail, Mahmoud Danaee, Rafiza Shaharudin

**Affiliations:** 1 Environmental Health Research Centre, Institute for Medical Research, National Institutes of Health, Ministry of Health, Setia Alam, Selangor 40170 Malaysia; 2 Clinical Research Centre, Hospital Sultanah Bahiyah, Alor Setar, Kedah 05460 Malaysia; 3 Department of Social and Preventive Medicine, Faculty of Medicine, University of Malaya, Kuala Lumpur, 50603 Malaysia

**Keywords:** Anthropometric survey, Face dimensions, Fit test panels, n-95, Respirators, Respirator sizing

## Abstract

**Background:**

The existing respiratory fit test panels (RFTPs) are based on Bivariate and Principal Component Analysis (PCA) which utilise American and Chinese head and facial dimensions. As RFTPs based on local facial anthropometric data for Malaysia are not available, this study was conducted with the aim to develop new RFTPs using Malaysian data.

**Methodology:**

A cross-sectional study was conducted across Malaysia among 3,324 participants of the study of National Health and Morbidity Survey 2020 aged 18 and above. Ten head and facial dimensions were measured. Face length and face width were used to construct bivariate facial panel, whereas the scores from the first two PCA were used to develop the PCA panel.

**Results:**

This study showed that Malaysians have the widest upper limit for facial width. It also found that three factors could be reduced from the PCA analysis. However only 2 factors were selected with PCA 1 representing head and facial size and PCA 2 representing facial shape. Our bivariate panel could accommodate 95.0% of population, while our PCA panel accommodated 95.6%.

**Conclusion:**

This was the first study to use Malaysian head and facial anthropometry data to create bivariate and PCA panels. Respirators constructed using these panels are likely to fit ≥ 95.0% of Malaysia’s population.

**Supplementary Information:**

The online version contains supplementary material available at 10.1186/s12890-024-02919-9.

## Background

Respirators are respiratory personal protective equipment (PPE) that protects the wearer from inhaling hazardous pollutants in the environment including fumes, vapours, and particulate matter such as dusts and airborne microbial pathogens. A respiratory fit test is required to ensure that respirators are comfortable to be used and to provide the expected level of protection. This is to minimise the total contaminant leakage into the facepiece through face seal. Prior to marketing, new models of respirators in Europe and the United States must meet certification requirements from National Institute Occupational Safety and Health (NIOSH) and the State Administration of Work Safety [1], in which the facepiece of the respirator is fit-tested on a panel of human subjects with facial sizes representative of the user population, which is traditionally the population in the nation or geographical area where the respirators are designed and manufactured. In this process, the respirator fit test panel (RFTP) is typically used as a matrix to select candidates to serve as representative test subjects [1].

There are currently four RFTPs that had been developed [1–4]. The first RFTP was developed in the 1960s by the Los Alamos National Laboratory (LANL) based on facial anthropometrics of personnels serving in the United States Air Force [2]. Two bivariate RFTPs were created by the LANL. The first bivariate RFTP was developed using the dimensions of menton-sellion length (face length) and bizygomatic breadth (face width) for fit testing full-face masks, and the second bivariate used dimensions of face length and lip length for fit testing half masks. Subsequently in 2003, the National Institute for Occupational Safety and Health (NIOSH) conducted a head-and-face anthropometric survey on 3997 United States civilian workers [1]. The findings of this large-scale anthropometric survey led to the development of a second RFTP with a bivariate panel using face length and face width, as well as a principal component analysis (PCA) panel using ten head and facial dimensions. The study revealed that the ten dimensions were associated with respirator fit and leakage. Therefore, respirators designed to fit these panels were expected to accommodate more than 95% of civilian workers of the United States.

It Is important to note that the RFTP developed by the NIOSH using head and facial dimensions from United States population may not be suitable for other populations. A study of 451 Chinese university students in China discovered that up to 35% of the participants were outside the ranges of the LANL fit test panels. This was because Chinese adults had shorter and wider facial characteristics than military personnel in the United States [5]. The correct fit of a respirator is determined by several factors, especially the head and facial dimensions. These dimensions have been shown to be affected by ethnicity, gender, age, body mass index (BMI), and geographic location [5–8]. Consequently, the third and fourth RFTPs were developed in China in 2006 [3] and Taiwan in 2016 [4], respectively.

Given that the existing bivariate and PCA panels are based on American and Chinese head and facial dimensions, and that no previous facial anthropometric research on RFTPs for Malaysia have been undertaken, this study aimed to fill this gap. The first part of our study, which focused on creating head and facial morphological database for Malaysia, has been published elsewhere [9]. In the present work, the purpose of this study was to develop new RFTP, including a bivariate panel and a PCA panel using Malaysian data.

## Methodology

### Malaysia anthropometric database

A population-based cross-sectional study was conducted across the country using complex sample design among participants in the National Health and Morbidity Survey (NHMS) 2020, aged 18 years and above. The sample strategy was discussed in full in the NHMS 2020 report [10] and in our previously published study [9]. In brief, the sampling frame for this study was based on the amended National Population and Housing Census 2020 by Department of Statistics Malaysia (DOSM) [11]. It included Malaysians from all 13 states and three Federal Territories. Stratified random sampling in two stages was used. The first stratum comprised all Malaysian states, while the second covered urban and rural areas within each state.

### Methods for data collection

The study employed direct measurement and analysis of 2D photogrammetry. Direct measurement using spreading calipers were used to measure head breadth, bizygomatic breadth and bigonial breadth, while 2D photogrammetry was used to measure the remaining seven dimension. The images of the participants were taken with a 20.0-megapixel digital camera. One anterior and one lateral photo were taken for each participant. The images were transferred to a computer. Digimizer version 5.4.4 was used to measure the anthropometric dimensions. Details of 2D photogrammetry methods can be found in previously published paper [9].

### Statistical analysis

The Statistical Package for Social Science SPSS Statistics (SPSS) version 26 and the R software version 4.2.3 were used for analysis. To obtain prevalence and population estimates with 95% confidence intervals, complex sample analysis with sample weight was used. Each sampled household’s weight would be the inverse of its selection probability (calculated by multiplying the probabilities at each sampling stage). The sample weights were calculated by adjusting the basic weight based on the non-response and post-stratification factors. Descriptive analysis for continuous data was presented as mean ± standard deviation (considering the dataset was large and normality was assumed), while categorical data were presented in frequency and column percentage.

The LANL and NIOSH used face length and face width as parameters in constructing the bivariate RFTP [1, 12]. To determine the limits of the bivariate panel, the upper limit was calculated using mean of males plus two standard deviations (SDs), and the lower limit was calculated using the mean of females minus two SDs. The boundaries were set with the aim of including at least 90% of the population in the bivariate panel. Following that, a bivariate RFTP was divided into ten size cells using the method described by Zhuang et al., and the cell boundaries were then adjusted to allow bivariate panel to capture 95% of the population and distribute it as uniformly as possible among cells [1]. In this study, these procedures were followed for the development of new bivariate RFTP based on the results of our anthropometric survey [9].

The head and face dimensions were grouped using PCA weighted to Malaysian population. PCA is a method of data reduction that statistically condense the information into a smaller number of dimensions, often called factors, without significantly reducing the amount of the information [13]. To determine the number of components to retain from the PCA, parallel analysis was conducted. Essentially, the program worked by creating a random dataset with the same number of observations and variables from the original data [14]. The numbers that were found to be higher than the simulated number were retained. However, to ensure the study was comparable to previous studies on PCA and bivariate panels [1, 3, 4], only the first two component analysis were retained, and the correlation tested using canonical correlation.

Next, factor loading for each dimension for PC 1 and PC 2 were converted to eigenvector. Eigenvector was equal to the factor loading of the dimension divided by the square root of eigenvalue of each factor. The eigenvector for each head and facial dimensions were used to multiply with the original 10 facial dimensions to obtain the scores for each PC.

Finally, the scores from the first two principal component analysis were used to develop the PCA panel. The panel was divided into 8-cell categories by dividing the eclipses into four quadrants by two lines. The inner and outer ellipse constant was set to include at least 50% and 95% of the population, respectively in the PCA panel.

### Patient and public involvement

The study participants were not involved in the development of this study. The results of the study were not shared with the participants.

## Results

This study comprised of 3,324 participants from all over Malaysia. There were 53.2% females and 46.7% males (Table 1). The mean age of study participants was 43.0 ± 16.2, with 82.5% of the study population being between the age of 18 and 60, and 17.5% being ≥ 61 years old. Malay ethnicity made up 64.0% of the study population, followed by Chinese (9.8%), Indian (5.3%) and other ethnicities (20.9%). 71.8% of our population was from west Malaysia and the remaining were from the east Malaysia. Details of the sociodemographic characteristics of our study participants were described further in our previously published paper [9].

**Table 1:** Sociodemographic data of the study participants

	Number (n)	Percentage (%)
Gender (*n* = 3324)		
Male	1556	46.7
Female	1768	53.2
**Age group** (*n* = 3324)		
18–60 years old	2741	82.5
> 60 years old	584	17.5
**Ethnicity** (*n* = 3324)		
Malay	2127	64.0
Chinese	326	9.8
Indian	176	5.3
Others	695	20.9
**Location** (*n* = 3324)		
East Malaysia	938	28.2
West Malaysia	2386	71.8

**Fig. 1:**
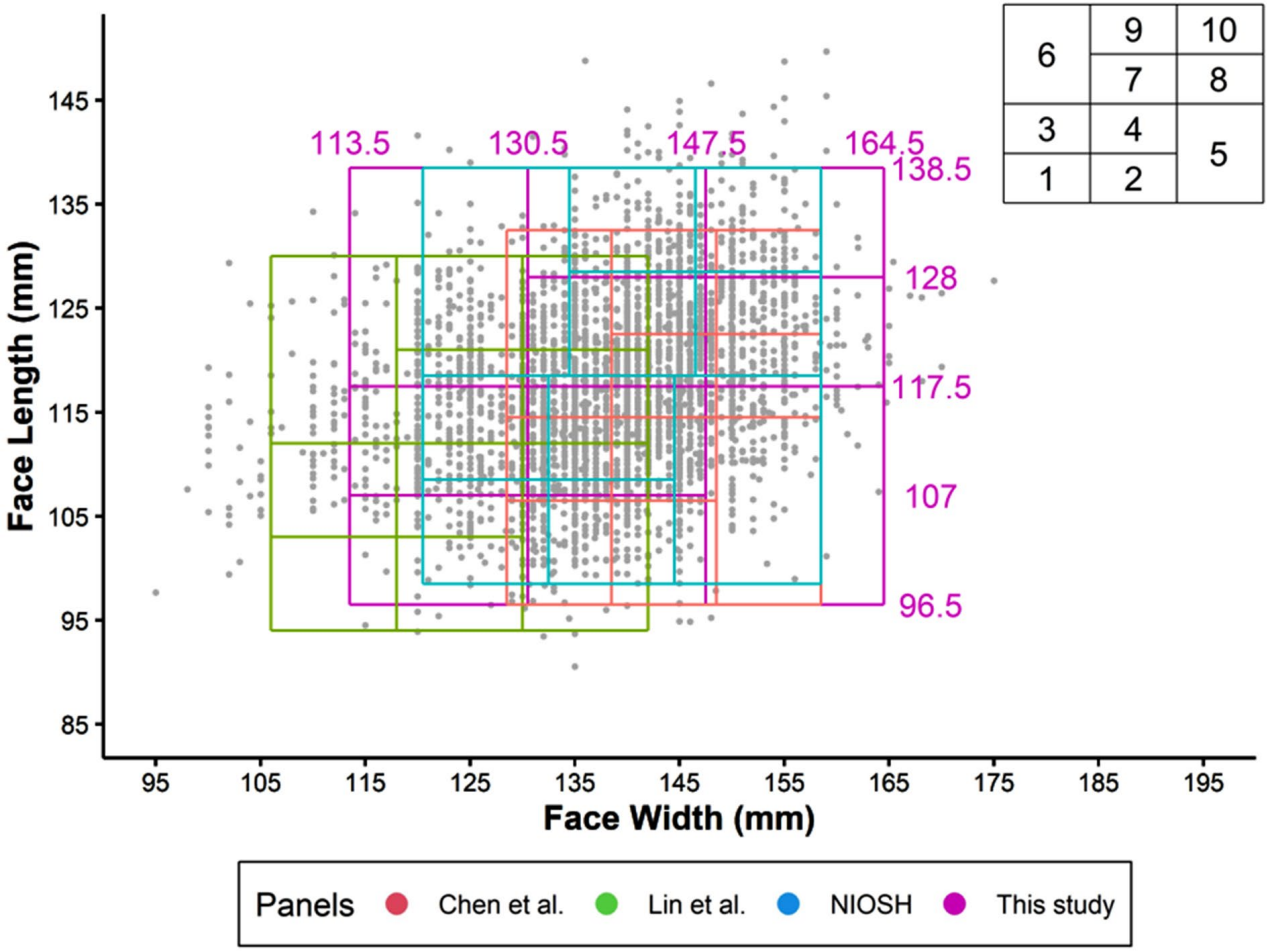
Head-and-face dimensions of participants from this study in bivariate panels of different RFTPs studies. References: Chen et al [3], Lin et al [4], NIOSH [1]

The bivariate fit test panel based on face length and face width of the local population in comparison to three previously published panels are showed in Fig. 1. Our local panel has limits of 96.5 to 138.5 mm for face length and 113.5 to 164.5 mm for face width. In comparison to fitting our local population into the bivariate panel created by NIOSH (89.2%), China (77.5%) and Taiwan (61.2%), the total number of participants included in our bivariate panel was the highest (95.0%), with 95.0% males and 95.0% females (Table 2**)**.

**Table 2:** Number and percentage Of participants from this study in bivariate panels Of different RFTPs

Panel	Cell	Male			Female			Total	
		n	(%)		n	(%)		n	(%)
NIOSH (2007)									
	1	14	(0.9)		117	(6.6)		131	(3.9)
	2	23	(1.5)		182	(10.3)		205	(6.2)
	3	68	(4.4)		242	(13.7)		310	(9.3)
	4	178	(11.4)		474	(26.8)		652	(19.6)
	5	152	(9.8)		209	(11.8)		361	(10.9)
	6	172	(11.1)		95	(5.4)		267	(8.0)
	7	353	(22.7)		184	(10.4)		537	(16.2)
	8	236	(15.2)		36	(2.0)		272	(8.2)
	9	107	(6.9)		25	(1.4)		132	(4.0)
	10	91	(5.8)		6	(0.3)		97	(2.9)
	**Total**	**1,394**	**(89.6)**		**1,570**	**(88.8)**		**2,964**	**(89.2)**
**Chen et al. (2009)**									
	1	9	(0.6)		115	(6.5)		124	(3.7)
	2	7	(0.4)		67	(3.8)		74	(2.2)
	3	48	(3.1)		309	(17.5)		357	(10.7)
	4	75	(4.8)		234	(13.2)		309	(9.3)
	5	29	(1.9)		51	(2.9)		80	(2.4)
	6	246	(15.8)		264	(14.9)		510	(15.3)
	7	252	(16.2)		219	(12.4)		471	(14.2)
	8	113	(7.3)		40	(2.3)		153	(4.6)
	9	257	(16.5)		67	(3.8)		324	(9.7)
	10	159	(10.2)		14	(0.8)		173	(5.2)
	**Total**	**1,195**	**(76.8)**		**1,380**	**(78.1)**		**2,575**	**(77.5)**
**Lin et al. (2017)**									
	1	1	(0.1)		2	(0.1)		3	(0.1)
	2	3	(0.2)		22	(1.2)		25	(0.8)
	3	10	(0.6)		32	(1.8)		42	(1.3)
	4	21	(1.3)		112	(6.3)		133	(4.0)
	5	50	(3.2)		429	(24.3)		479	(14.4)
	6	31	(2.0)		46	(2.6)		77	(2.3)
	7	60	(3.9)		116	(6.6)		176	(5.3)
	8	239	(15.4)		443	(25.1)		682	(20.5)
	9	64	(4.1)		25	(1.4)		89	(2.7)
	10	222	(14.3)		106	(6.0)		328	(9.9)
	**Total**	**701**	**(45.1)**		**1,333**	**(75.4)**		**2,034**	**(61.2)**
**For this study**									
	1	11	(0.7)		74	(4.2)		85	(2.6)
	2	20	(1.3)		183	(10.4)		203	(6.1)
	3	67	(4.3)		231	(13.1)		298	(9.0)
	4	225	(14.5)		655	(37.0)		880	(26.5)
	5	75	(4.8)		86	(4.9)		161	(4.8)
	6	146	(9.4)		93	(5.3)		239	(7.2)
	7	478	(30.7)		283	(16.0)		761	(22.9)
	8	220	(14.1)		37	(2.1)		257	(7.7)
	9	141	(9.1)		30	(1.7)		171	(5.1)
	10	95	(6.1)		8	(0.5)		103	(3.1)
	**Total**	**1,478**	**(95.0)**		**1,680**	**(95.0)**		**3,158**	**(95.0)**

*References* Chen et al [3], Lin et al [4], NIOSH [1]

**Fig. 2:**
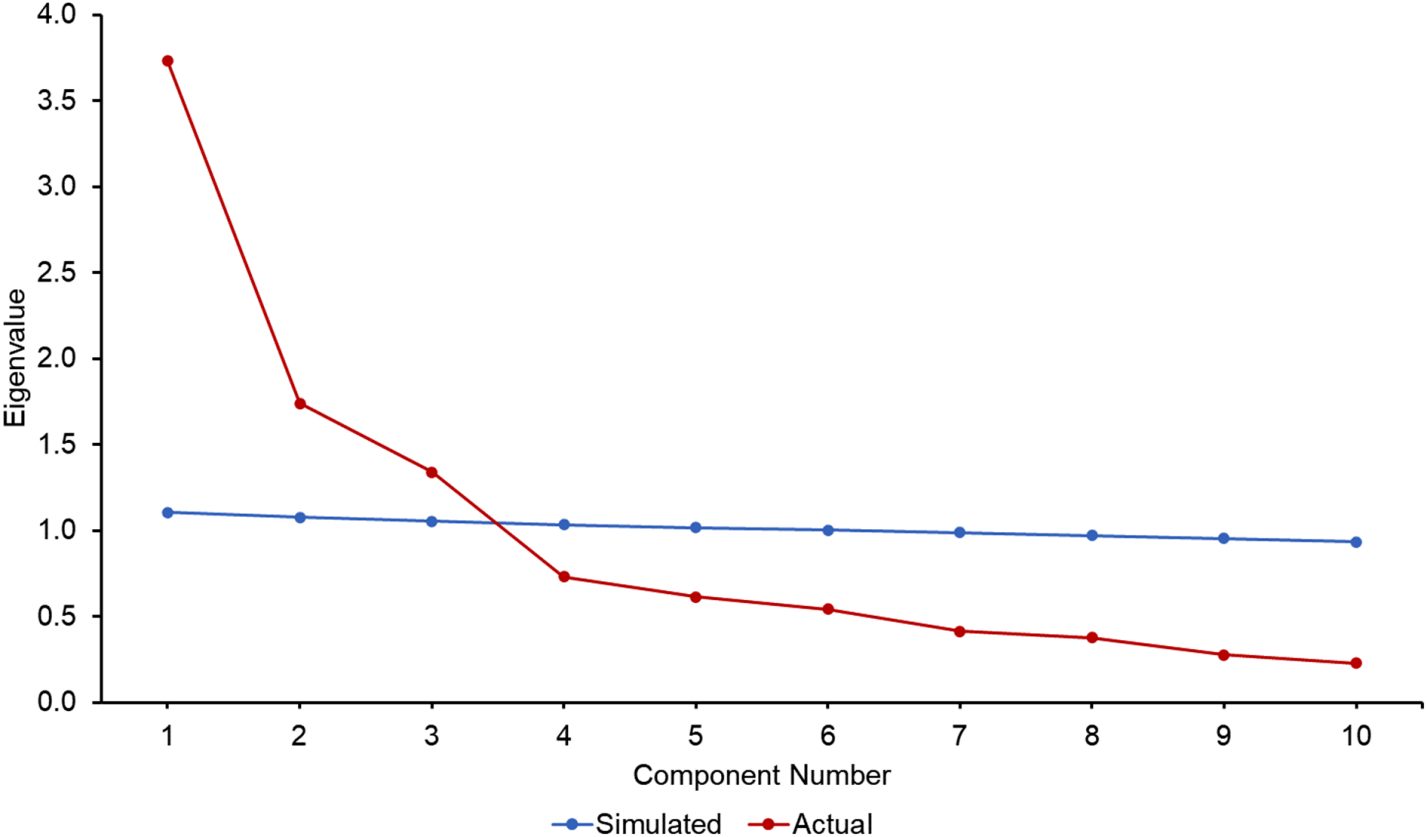
Scree plot of Malaysia PCA analysis

Table 3 shows the results of PCA weighted to Malaysian population. There were 10 items in the construct with 3 main factors extracted, with the first PCA explaining 37.4% of variances, followed by the second and third factors explaining 17.4% and 13.4%, respectively. It was worthy to note that these three factors could explain 68.2% of the total variances. The result was supported by the scree plot (Fig. 2), in which the slope from Factor 1 to 3 was very steep and parallel analysis indicated that the eigenvalue of these three factors have more than the eigenvalue of simulated data (44.1%). Hence, most of the variances could be explained by Factor 1 to 3. From Factor 4 onwards, the slopes were very gentle. This showed that the subsequent Factors (Factor 4 to 10) did not contribute much to the explained variance.

**Table 3:** Principal component analysis weighted To Malaysian population

Principal Component	Eigenvalues	Cumulative Eigenvalues	Total Variance (%)	Cumulative Variance (%)
1	3.735	3.735	37.4	37.4
2	1.739	5.474	17.4	54.8
3	1.340	6.813	13.4	68.2
4	0.733	7.547	7.3	75.5
5	0.615	8.161	6.1	81.6
6	0.543	8.705	5.4	87.0
7	0.414	9.119	4.1	91.1
8	0.377	9.496	3.8	94.9
9	0.276	9.771	2.8	97.7
10	0.229	10.000	2.3	100.0

However, to ensure that our result was comparable with the previous studies [1, 3, 4] only two principal components were chosen, namely PC 1 and PC 2. The results showed that head breadth, minimum frontal breadth, interpupillary distance, bizygomatic breadth, menton-sellion length, bigonial breadth, nasal root breadth and nose breadth loaded heavily on PC 1 while subnasale-sellion length and nose protrusion loaded highly on PC 2 (Table 4). Based on this grouping, PC 1 can be summarised as head and facial size, PC 2 as facial shape. The canonical correlation value between the two factors was 0.529, which was more than 0.3, indicating PC1 and PC2 are interrelated.

**Table 4:** Component matrix with two principal component extracted To Malaysian population

Facial Dimensions	Principal Component 1	Principal Component 2
Head breadth	0.621452	-0.444311
Minimum frontal breadth	0.618769	-0.157589
Interpupillary distance	0.722038	-0.060907
Bizygomatic breadth (face width)	0.715528	-0.433634
Menton-sellion length (face length)	0.660795	0.564092
Bigonial breadth	0.623320	-0.366189
Nasal root breadth	0.458948	-0.082074
Nose breadth	0.679237	0.019471
Subnasale-sellion length (nose length)	0.451620	0.725292
Nose protrusion	0.481299	0.582327

The eigenvectors for PC 1 were all positive (Table 5). The top three dimensions that contributed the most to PC 1 were interpupillary distance, bizygomatic breadth and nose breadth. On the other hand, there were a combination of positive (menton-sellion length, nose breadth, subnasale-sellion length and nose protrusion) and negative eigenvectors (head breadth, minimum frontal breadth, interpupillary distance, bizygomatic breadth, bigonial breadth and nasal root breadth) in the PC 2. The top three dimensions contributing to PC 2 in this study were menton-sellion length, subnasale-sellion length and nose protrusion.

**Table 5:** Comparison of eigenvectors divided into two principal component analysis in the current and previous studies

Head-and-face dimension	First principal component					Second principal component			
	Current study	NIOSH	Chen et al.	Lin et al.		Current study	NIOSH	Chen et al.	Lin et al.
Head breadth	0.321552	0.372241	0.373045	0.641031		-0.336971	0.013306	-0.132683	0.032379
Minimum frontal breadth	0.320164	0.343264	0.322260	0.555771		-0.119517	-0.152951	-0.388836	-0.434911
Interpupillary distance	0.373598	0.363474	0.370307	0.766379		-0.046193	-0.173099	-0.159748	-0.309213
Bizygomatic breadth (face width)	0.370229	0.426498	0.422051	0.665253		-0.328873	-0.039087	-0.140757	-0.084469
Menton-sellion length (face length)	0.341909	0.329648	0.244826	0.644739		0.427814	0.359799	0.568632	0.526096
Bigonial breadth	0.322519	0.372717	0.328562	0.513814		-0.277722	-0.093279	-0.227790	-0.060759
Nasal root breadth	0.237469	0.202311	0.159204	0.569534		-0.062246	-0.341235	-0.173192	-0.294401
Nose breadth	0.351452	0.301125	0.321181	0.712228		0.014767	-0.210833	0.079405	-0.071652
Subnasale-sellion length (nose length)	0.233678	0.193650	0.297905	0.426506		0.550071	0.584261	0.528574	0.724967
Nose protrusion	0.249034	0.113578	0.237882	0.469558		0.441644	0.551842	0.308739	0.246291

*References* Chen et al [3], Lin et al [4], NIOSH [1]

The eigenvectors for each head and facial dimensions were multiplied by the original 10 facial dimensions to obtain the scores for each PC. The PC 1 and PC 2 were then calculated as follows:

**PC 1** = 0.321552*(head breadth) + 0.320164*(minimum frontal breadth) + 0.373598*(interpupillary distance) + 0.370229*(face width) + 0.341909*(face length) + 0.322519*(bigonial breadth) + 0.237469*(nasal root breadth) + 0.351452*(nose breadth) + 0.233678*(subnasale-sellion length) + 0.249034*(nose protrusion).

**PC 2** = − 0.336971*(head breadth) − 0.119517*(minimum frontal breadth) − 0.046193*(interpupillary distance) − 0.328873*(face width) + 0.427814*(face length) − 0.277722*(bigonial breadth) − 0.062246*(nasal root breadth) + 0.014767*(nose breadth) + 0.550071*(subnasale-sellion length) + 0.441644*(nose protrusion).

**Fig. 3:**
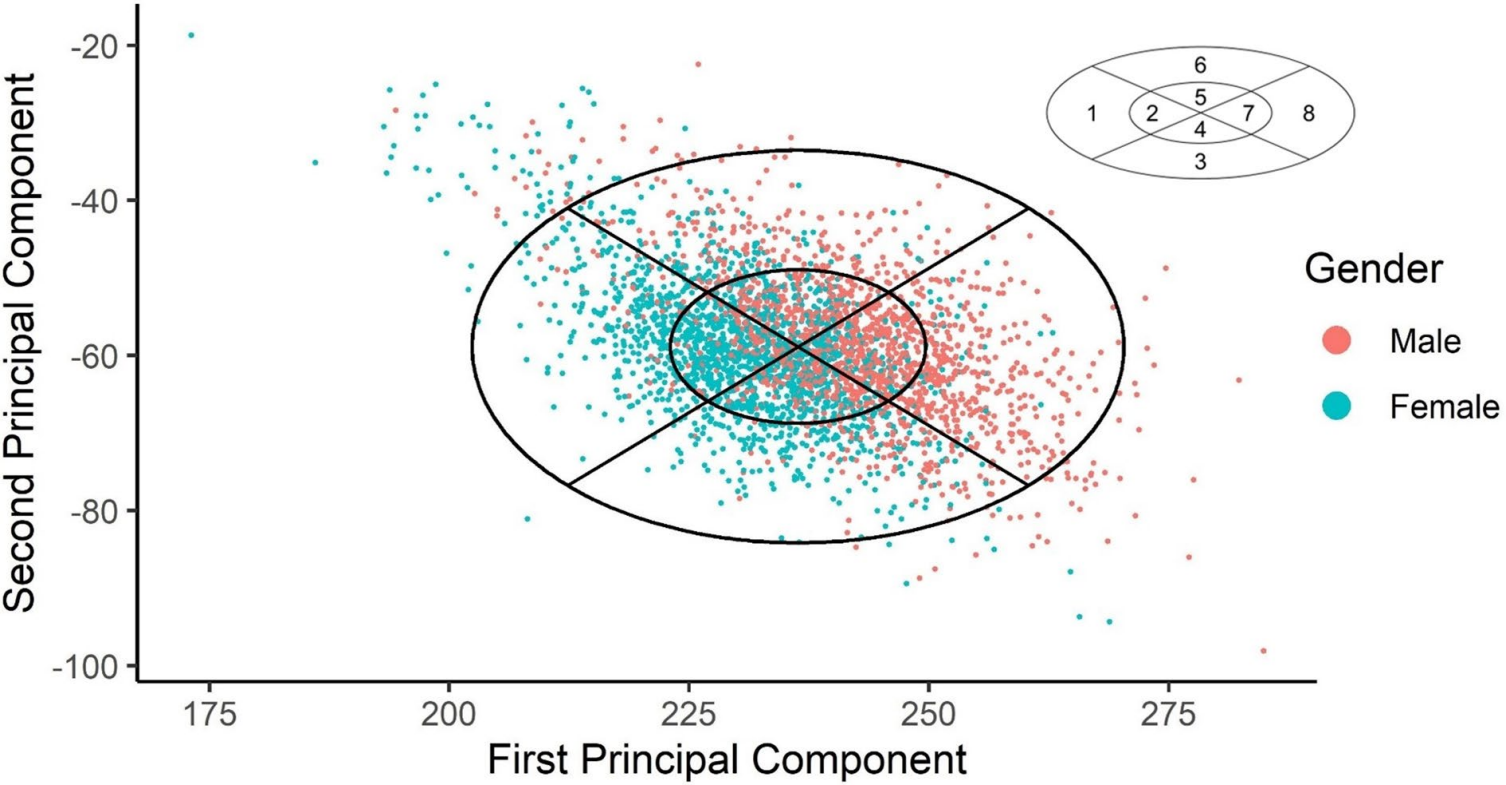
Scatter plot of principal components scores shown along the Malaysian PCA panel

Fig. 3 shows fit test panel utilising the scatter plot of the PC scores. The limit of this panel was based on an ellipse in which more than 95% of the population was included. The outer ellipse contained 95.6% whereas the inner ellipse contained 54.1% of the population. The means ± SD for PC 1 and PC 2 were 265.64401 ± 13.56653 and − 57.62220 ± 9.12037 respectively, with constants of 1.095 for inner ellipse (Supplementary material Appendix A). Our study showed a positive correlation between PCA 1 and PCA, which was consistent with the study conducted in China, but a negative correlation was found in studies conducted by NIOSH and Taiwan (Supplementary material e-Fig. 1).

The number and the percentage of the local population that fits into previously developed PCA of other countries and our newly developed local PCA is showed in Table 6. The overall number of participants included in our newly developed PCA was the greatest (95.6%), compared to fitting our local population into the PCA panel prepared by NIOSH (92.6%), China (75.4%), and Taiwan (78.2%). Our PCA can accommodate 94.8% males and 96.1% females. The distributions of the cells are relatively uniform, with the percentage ranging from 9.4 to 14.7%.

**Table 6:** Number and percentage of participants from this study in principal component analysis panels of different RFTPs studies

Panel	Cell	Male			Female			Total	
		n	(%)		n	(%)		n	(%)
NIOSH (2007)									
	1	76	(4.9)		309	(17.5)		385	(11.6)
	2	145	(9.3)		287	(16.2)		432	(13.0)
	3	186	(12.0)		457	(25.8)		643	(19.3)
	4	225	(14.5)		329	(18.6)		554	(16.7)
	5	179	(11.5)		93	(5.3)		272	(8.2)
	6	116	(7.5)		41	(2.3)		157	(4.7)
	7	266	(17.1)		76	(4.3)		342	(10.3)
	8	264	(17.0)		28	(1.6)		292	(8.8)
	**Total**	**1,457**	**(93.8)**		**1,620**	**(91.6)**		**3,077**	**(92.6)**
**Chen et al. (2009)**									
	1	41	(2.6)		393	(22.2)		434	(13.1)
	2	51	(3.3)		166	(9.4)		217	(6.5)
	3	16	(1.0)		56	(3.2)		72	(2.2)
	4	18	(1.2)		60	(3.4)		78	(2.3)
	5	172	(11.1)		187	(10.6)		359	(10.8)
	6	582	(37.4)		432	(24.4)		1,014	(30.5)
	7	98	(6.3)		43	(2.4)		141	(4.2)
	8	179	(11.5)		14	(0.8)		193	(5.8)
	**Total**	**1,157**	**(74.4)**		**1,351**	**(76.4)**		**2,508**	**(75.4)**
**Lin et al. (2017)**									
	1	27	(1.7)		140	(7.9)		167	(5.0)
	2	17	(1.1)		174	(9.8)		191	(5.7)
	3	11	(0.7)		74	(4.2)		85	(2.6)
	4	21	(1.3)		114	(6.4)		135	(4.1)
	5	103	(6.6)		268	(15.2)		371	(11.2)
	6	425	(27.3)		488	(27.6)		913	(27.5)
	7	92	(5.9)		164	(9.3)		256	(7.7)
	8	343	(22.0)		135	(7.6)		478	(14.4)
	**Total**	**1,039**	**(66.6)**		**1,557**	**(88.0)**		**2,596**	**(78.2)**
**For this study**									
	1	38	(2.4)		301	(17.0)		339	(10.2)
	2	78	(5.0)		409	(23.1)		487	(14.7)
	3	118	(7.6)		218	(12.3)		336	(10.1)
	4	167	(10.7)		323	(18.3)		490	(14.7)
	5	223	(14.3)		162	(9.2)		385	(11.6)
	6	189	(12.1)		124	(7.0)		313	(9.4)
	7	313	(20.1)		119	(6.7)		432	(13.0)
	8	352	(22.6)		45	(2.5)		397	(11.9)
	**Total**	**1,478**	**(94.8)**		**1,701**	**(96.1)**		**3,179**	**(95.6)**

*References* Chen et al [3], Lin et al [4], NIOSH [1]

## Discussion

Two RFTPS were generated using the local data, namely a bivariate panel and a RFTP panel. The finding of this study revealed that the boundaries for facial size and length differed from previous studies. The panel of the current study had limits of 96.5 to 138.5 mm for face length and 113.5 to 164.5 mm for face width. These limits differed from the NIOSH’s bivariate panel limits of 98.5 to 138.5 mm for face length and 120.5 to 158.5 mm for face width, study in Taiwan has the limit of 90 to 131 mm for face length and 105 to 145 mm for face width, as well as the Chinese bivariate panel of 96.5 to 132.5 mm for face length and 128.5 to 158.5 mm for face width. It was worth noting that we have the widest face width compared to others. The current study has same lower limit of face length as the Chinese study and the same upper limit of face length as the study by NIOSH.

Among the three previously developed bivariate panels and PCA panels, the panels developed NIOSH achieved the highest fitting for both bivariate panel (89.2%) and for PCA panel (92.6%). The study conducted from Taiwan had the lowest matching for the bivariate panel (61.2%), and the study from China had lowest matching for PCA (75.4%). The reason for the low matching could be due to the differences in the head and facial dimensions of population from different continents. As a result, there is a need to develop our panels with the aim to accommodate 95% of the local population. The bivariate panel generated by this study could accommodate 95.0% of the local population whereas the PCA panel could accommodate up to 95.6%.

In order to ensure that findings of this study were comparable to previous studies, we used the first two principal components for further analysis. In our study, the first two major components explained 54.8% of the total variance. Our results were lower than the study conducted by NIOSH (58.5%), but higher than the studies conducted in China (52.7%) and Taiwan (49.1%) [1, 3, 4]. We removed the third principal component, which had contributed up to 14.9% of the variance. The reason was because direct comparisons with the prior PCA panels became challenging as more than two PCs were preserved, and thus making the scatter plots difficult to understand. The same methodology using two PCs was applied in the NIOSH study (eliminating the 3rd principal component which contributed to 8.6% of the variance), the China study (eliminating the 3rd principal component which contributed to 12.1% of the variance), and the Taiwan study (eliminating the 3rd principal component which contributed to 10.6% of variance) [1, 3, 4].

In our study, the top three dimensions that contributed the most to PC 1 were interpupillary distance, bizygomatic breadth, and nose breadth. The only dimension shared by these four RFTPs studies in the top three dimensions in PC 1 was bizygomatic breadth. On the other hand, the top three dimensions contributing to PC 2 in our study were nose breadth, nose protrusion and subnasale-sellion length, which was consistent with previous studies [1, 3, 4]. Our PC showed positive correlation between PC 1 and PC 2, which was in line with the findings of the Chinese study. However, the studies by NIOSH and Taiwan found a negative correlation. The main reason for this was that the positive and negative direction of eigenvectors of the measured dimension for PC 2 were not the same across studies.

This study had some limitations. Earlier studies on RFTPs used purely direct measurement techniques whereas our study used a combination of direct and 2D measurement methods. However, it should be noted that 2D photogrammetry was discovered to be a validated tool in our previous published study [15], which was supported by many other previously published papers [16–21]. Another limitation of the current study is that, because there have been no previous studies [1, 3–5] that grouped the dimensions into three factors, the third factor from the analysis was excluded to make sure our study is comparable with other studies. Another limitation is that the current study only measured ten dimensions, as opposed to previous studies that measured up to 18 measurements [6, 7]. The top ten measurements contributing to the fit test panel were determined using multiple regression analyses, which were not performed here. Lastly, the negative correlation between PC 1 and PC 2, in contrast to prior studies [1, 4] remained unexplained.

The main strength of current study was that it is the first large nationwide study on head and facial anthropometry in Malaysia with 3,324 participants [9]. Another strength of the current study was its use of complex analysis with weighted sample, which was important for improving the precision of sample estimates and could be used to account for non-responses and non-coverage.

## Conclusion

The findings of the present study are important for Malaysia, given that this is the first study to produce our own bivariate and PCA panel according to the head and facial anthropometry of Malaysian population, whereby the respirator designed to fit these panels are likely to accommodate more than 94% of the Malaysian population.

### Electronic supplementary material

Below is the link to the electronic supplementary material.

**Supplementary material 1 (Appendix A):** Algorithm for classifying test subjects into the PCA panel cells

**Supplementary material 2 (e-Figure 1):** Head-and-face dimensions of participants from this study in Principal Component Analysis Panels of different RFTPs studies

## Data Availability

The datasets used and/or analysed during the current study are available from the corresponding author on reasonable request.

## Abbreviations

RFTPs: respiratory fit test panels
PCA: Principal Component Analysis
NIOSH: National Institute Occupational Safety and Health
LANL: Los Alamos National Laboratory
PPE: personal protective equipment
BMI: body mass index
DOSM: Department of Statistics Malaysia
SPSS: Statistical Package for Social Science SPSS Statistics
