# Supplementary material for: Respiratory fit test panel representing population of Malaysia
Source: BMC Pulm Med. 2024 Mar 7;24:122. doi: 10.1186/s12890-024-02919-9 (PMC10921698; doi:10.1186/s12890-024-02919-9)
Supplement: Supplementary file 1 — Supplementary material 1 (Appendix A): Algorithm for classifying test subjects into the PCA panel cells [file 12890_2024_2919_MOESM1_ESM.docx]

**APPENDIX A**

**Algorithm for Classifying Test Subjects into the PCA Panel Cells**

PC1 = 0.321552*(head breadth) + 0.320164*(minimum frontal breadth) + 0.373598*(interpupillary distance) + 0.370229*(face width) + 0.341909*(face length) + 0.322519*(bigonial breadth) + 0.237469*(nasal root breadth) + 0.351452*(nose breadth) + 0.233678*(subnasale-sellion length) + 0.249034*(nose protrusion)

PC2 = − 0.336971*(head breadth) − 0.119517*(minimum frontal breadth) − 0.046193*(interpupillary distance) − 0.328873*(face width) + 0.427814*(face length) − 0.277722*(bigonial breadth) − 0.062246*(nasal root breadth) + 0.014767*(nose breadth) + 0.550071*(subnasale-sellion length) + 0.441644*(nose protrusion)

PC1 mean = 265.64401; SD1 = 13.56653.

PC2 mean = -57.62220; SD2 = 9.12037.

*x* = PC1 − 265.64401

*y* = PC2 + 57.62220

slope = 9.12037/13.56653 = 0.6722699

**Panel with more than 95% in gender population**

*a* = 2.770 * 13.56653

*b* = 2.770 * 9.12037

*c* = 1.095 * 13.56653

*d* = 1.095 * 9.12037

*r*_1_ = sqrt ((*x*^2^)/(*a*^2^)+ (*y*^2^)/(*b*^2^))

*r*_2_ = sqrt ((*x*^2^)/(*c*^2^)+ (*y*^2^)/(*d*^2^))


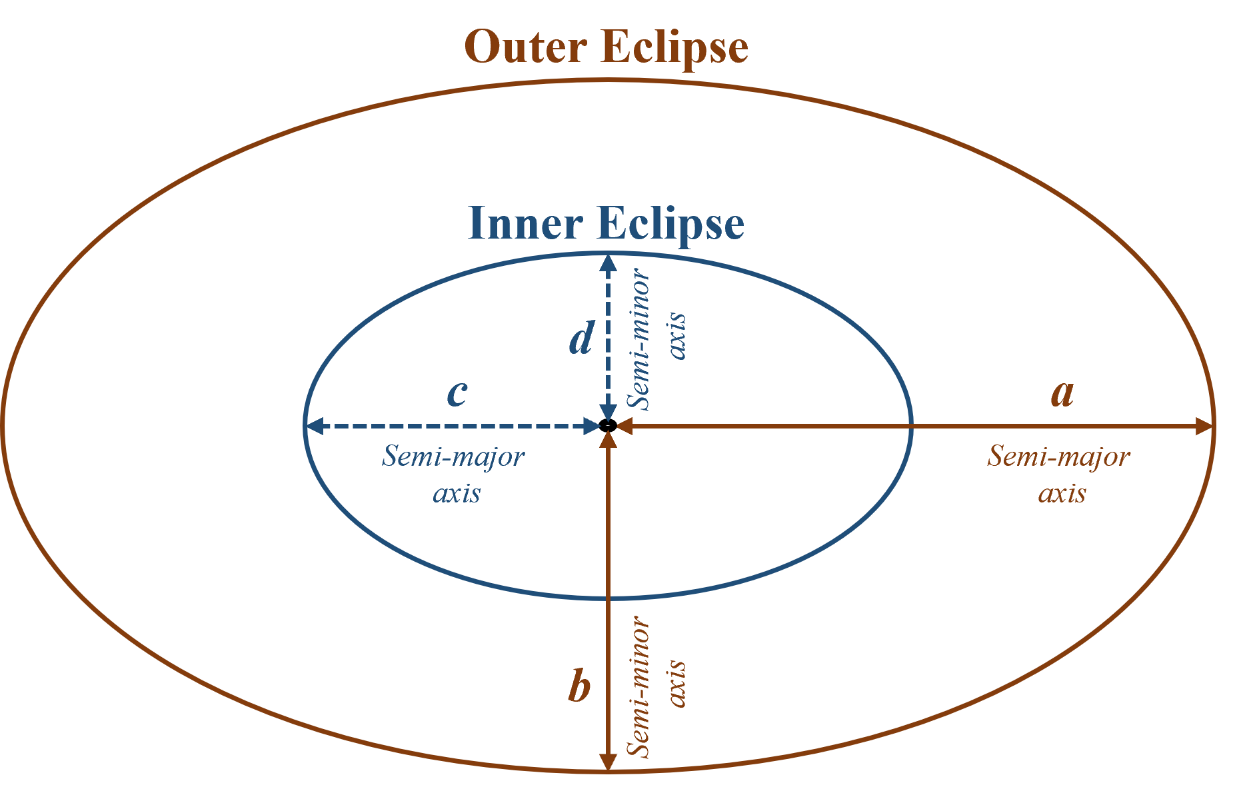


Use the *x*, *y* and *r*_1_ values and the algorithm below to determine if the subject is in cells 1, 3, 6 and 8:

| ***x*** | ***y*** | ***r*_1_** | **abs(*y*)/abs(*x*)** | **Cell** |
| --- | --- | --- | --- | --- |
| ≥ 0 | ≥ 0 | ≤ 1 | ≤ slope | 8 |
| ≥ 0 | < 0 | ≤ 1 | < slope | 8 |
| ≥ 0 | < 0 | ≤ 1 | ≥ slope | 3 |
| < 0 | < 0 | ≤ 1 | > slope | 3 |
| < 0 | < 0 | ≤ 1 | ≤ slope | 1 |
| < 0 | ≥ 0 | ≤ 1 | < slope | 1 |
| < 0 | ≥ 0 | ≤ 1 | ≥ slope | 6 |
| ≥ 0 | ≥ 0 | ≤ 1 | > slope | 6 |

If the *r*_2_ value is less than or equal to 1, use the following algorithm to adjust the cell number:

| **Cell** | ***r_2_*** | **Adjust Cell** |
| --- | --- | --- |
| 8 | ≤ 1 | 7 |
| 3 | ≤ 1 | 4 |
| 1 | ≤ 1 | 2 |
| 6 | ≤ 1 | 5 |
