# Supplementary material for: Respiratory fit test panel representing population of Malaysia
Source: BMC Pulm Med. 2024 Mar 7;24:122. doi: 10.1186/s12890-024-02919-9 (PMC10921698; doi:10.1186/s12890-024-02919-9)
Supplement: Supplementary file 2 — Supplementary material 2 (e-Figure 1): Head-and-face dimensions of participants from this study in Principal Component Analysis Panels of different RFTPs studies [file 12890_2024_2919_MOESM2_ESM.docx]

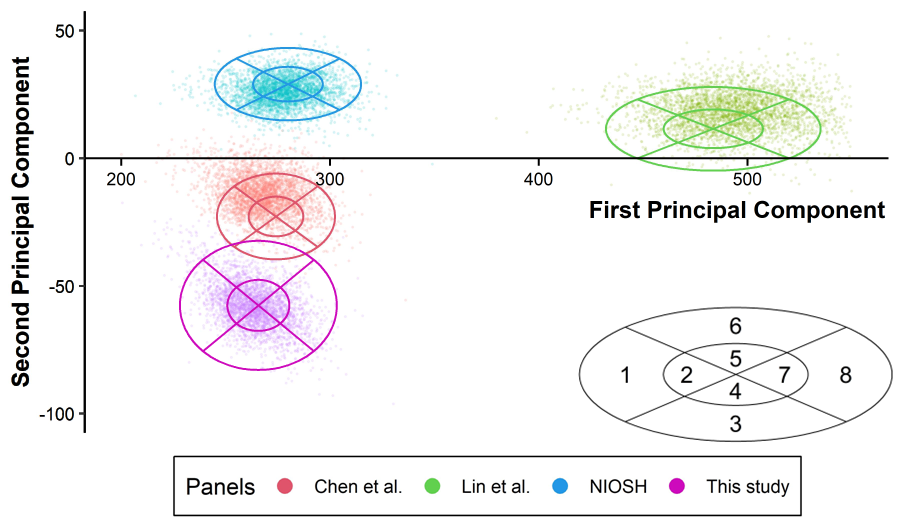


**e-Figure 1:** **Head-and-face dimensions of participants from this study in Principal Component Analysis Panels of different RFTPs studies.**

References: Chen et al(3), Lin et al(4), NIOSH(1)
